# Successful treatment of bronchial obstruction by flexible bronchoscopy and isoniazid: A case report

**DOI:** 10.3892/etm.2013.1423

**Published:** 2013-11-25

**Authors:** HENGYI CHEN, XIN HONG, YONG HE

**Affiliations:** Department of Respiratory Medicine, Daping Hospital, The Third Military Medical University, Chongqing 400042, P.R. China

**Keywords:** interventional bronchologists, isoniazid, bronchial obstruction

## Abstract

Interventional bronchology techniques have been employed as an effective first-line treatment in patients with tracheobronchial obstruction. However, recurrent stenosis produced by granulation tissue requires repeated procedures. Previous studies have indicated that isoniazid regulates collagen deposition and decreases collagen content. Thus, isoniazid has been successfully administered to patients with lesions who exhibited a delay in the healing process. A case of a left mainstem obstruction managed by interventional bronchology is described in the present study. Repeated bronchial stenosis was observed even following numerous treatment procedures, however, administration of isoniazid resulted in the inhibition of hypertrophic scar formation and prevention of repeated stenosis. The suppressive effect of isoniazid on granulation formation and further observations are reported. Few studies have been conducted with regard to the function of isoniazid in suppressing scar hyperplasia, therefore, the mechanism requires further investigation.

## Introduction

Benign tracheobronchial obstructions, arising from trauma or tumours, result in a persistent cough, asthma-like wheezing, dyspnea and ventilatory failure. These obstructions significantly affect quality of life and therefore, appropriate treatment is required. Although the successful treatment of tracheal stenosis using steroid regimens has been demonstrated (1), the mainstay of treatment for symptomatic lesions is surgery, including sleeve resection (2,3), however this is not without risk to the patient. Interventional bronchology techniques, including argon plasma coagulation (APC), cryotherapy, tracheal dilation and stenting, are widely employed as effective treatments for patients with central airway obstruction or less serious lesions (4,5). The choice of therapy depends on tumour size, pathology, wall invasion depth, anatomical location, degree of symptoms, patient co-morbidities and operator experience and expertise (6). However, the occurrence of repeated stenosis is relatively frequent, which is the principal long-term problem of interventional bronchology techniques (7).

## Case report

A 36-year-old male was admitted to Daping Hospital (Chongqing, China) following a workplace accident. A chest radiograph exhibited multiple rib fractures and lung contusions. The patient underwent surgery and was readmitted to hospital one month later due to difficulty breathing. The patient had no history of chronic lung disease or cyanosis and no related medical history. This study was conducted in accordance with the declaration of Helsinki and was approved by the Ethics Committee of the Third Military Medical University (Chonqing, China). The participant provided written informed consent for involvement in this study.

**Figure 1:**
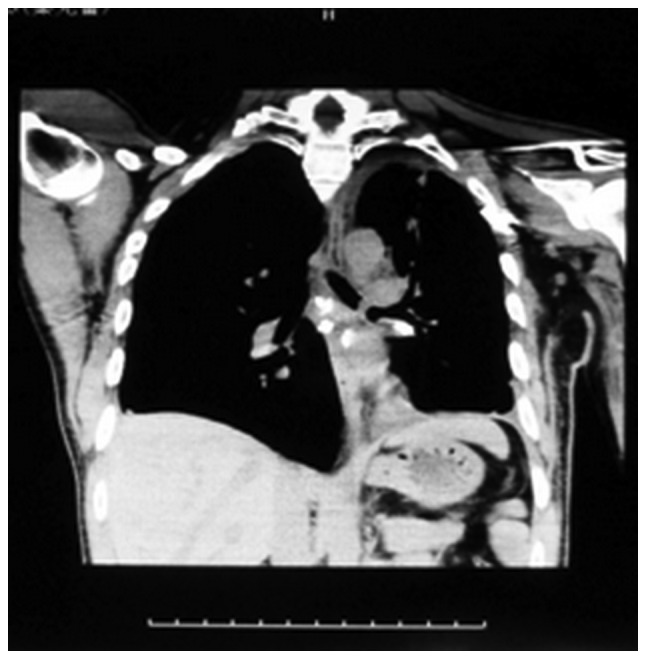
Coronal view of the computed axial tomography angiogram showing total occlusion of the left mainstem bronchus.

**Figure 2:**
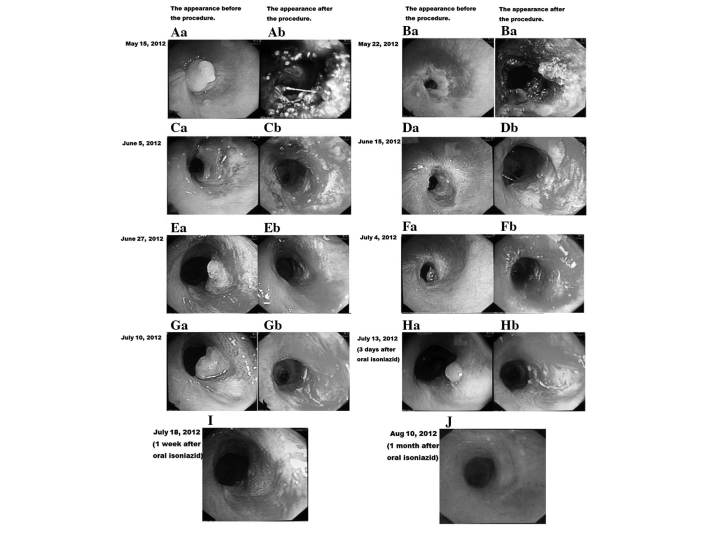
(Aa) A pedicle neoplasm with complete occlusion of the left mainstem bronchus prior to the procedure. (Ba, Bb, Ca, Cb, Da, Db, Ea, Eb, Fa, Fb, Ga and Gb) The left mainstem bronchus was narrowed by fibrous tissue obscuring the stent following repetitive procedures. (Ha, Hb, I and J) Restenosis was repressed following administration of oral isoniazid.

A physical examination revealed reduced breath sounds and mild wheezing in the left zone. A computed axial tomography angiogram of the chest demonstrated total occlusion of the left mainstem bronchus due to a soft tissue mass and atelectasis of the left upper lobe (Fig. 1). The vocal cords, trachea, carina and right bronchial tree were observed to be normal and flexible bronchoscopy was performed. A pedicle neoplasm with complete occlusion of the left mainstem bronchus was confirmed (Fig. 2Aa). The neoplasm was biopsied and the specimen was sent for histological examination, revealing inflammatory infiltration and fibrosis.

Rigid forceps were initially used to remove the granulation tissue in order to avoid surgery and produce rapid palliation of dyspnea. However, 70% of the airway remained obstructed due to the residual granulation tissue. Thus, APC and cryotherapy were utilised to devitalise and remove the residual granulation tissue surrounding the stenosis. This intervention reopened the obstructed left mainstem bronchus (Fig. 2Ab) and residual stenosis was estimated to be <20%. The procedure took 60 min.

One week later, the dyspnea symptoms reappeared and stenosis of the left mainstem bronchus was observed. APC and cryotherapy were performed 15 times as the left mainstem bronchus narrowed due to fibrous tissue obscuring the stent (Fig. 2Ba, Bb, Ca, Cb, Da, Db, Ea, Eb, Fa, Fb, Ga and Gb). By following related previous studies (8,9), isoniazid may regulated collagen deposition by inhibiting lysyl oxidase (LOX), so it was possible to avoid repeated and prolonged application of bronchoscopy with isoniazid. Oral isoniazid (~0.3g) (Shanghai Xinyi Pharmaceutical Co., Ltd., Shanghai, China) was administered once daily, prior to breakfast, for two weeks. On the third day, flexible bronchoscopy revealed a small granulation nodule on the sidewall of the trachea; this was frozen and removed by cryotherapy (Fig. 2Ha and Hb). Repeat flexible bronchoscopy was performed one week and one month following oral isoniazid administration (Fig. 2I and J). No recurrence of bronchial stenosis was observed. In addition, isoniazid appeared to be safe and did not result in any detectable liver lesions or renal damage. The patient was followed up for one year and no tracheal narrowing was observed.

## Discussion

Benign tracheobronchial obstruction in adults commonly result from tracheotomy procedures, intubation trauma or following blunt chest trauma (10). Additional causes include severe inflammation, autoimmune disorders and inhalation injuries. Tracheobronchial stenosis, arising from benign or malignant diseases, is often associated with dyspnea, ventilatory failure and obstructive pneumonia. If hypertrophic and keloid scarring are not actively treated they may lead to more serious stenosis or obstruction. Segmental and lobar atelectasis may emerge and result in suffocation due to airway obstruction. Bobocea *et al*(3) hypothesised that bronchial rupture as a result of blunt chest trauma (involving chest wall compression, traction on the carina and the sudden increase in intraluminal pressure) leads to granulation tissue formation that may result in progressive bronchial obstruction with distal infection and permanent parenchymal damage.

The optimal treatment for tracheobronchial obstructions remains undefined. Previously, patients with central airway obstructions were managed by surgery; however, for tracheobronchial obstruction, surgery is complex and may be traumatic (11). Furthermore, numerous patients are unsuitable for surgery as a result of poor lung function. In addition, specific patients may be affected by recurrent stenosis due to scarring and granulation tissues at the surgical site (12). With the development of interventional pulmonology in the last 20 years, interventional bronchology techniques, including neodymium-yttrium-aluminium-garnet laser irradiation, electrocautery, APC, cryotherapy, tracheal dilatation and airway stenting (10,13) allow for palliative techniques that alleviate central airway obstruction (14). As a single method is not able to solve all clinical issues, various interventional modalities are frequently combined to reduce complications and enhance the duration of the treatment effect (15). The choice of therapy is often dependent on the preference of the physician and local resources.

Management of a left bronchial obstruction malignant lesion using a combination of therapies, including APC with cryotherapy, was observed in this case study. APC produces a discontinuous current between the probe and tissue by iodinating argon, which exhibits a homeostatic effect. Cryotherapy freezes and leads to necrosis of the neoplasm, does not induce hypoxemia, lowers the risk of perforation, is not flammable and does not produce fumes. APC combined with cryotherapy, as applied in this case, rapidly reopened the occluded airway, reduced surgical duration and prevented severe haemorrhaging (16). However, the repeated stenosis of the opened airway due to scarring and subepithelial fibrosis was problematic, thus, oral isoniazid was administered for two weeks as a fibroblast inhibitor. No granulation or scar tissue was observed one month and one year following the procedure. Furthermore no adverse reactions were observed suggesting that isoniazid therapy is safe.

Previous studies have revealed that mitomycin, corticosteroid and 5-fluorouracil also reduce the recurrence rate of stenosis (17,18). However, these drugs are complex to administer in clinical applications due to significant toxicity, including myelosuppression. Carrington *et al*(8) observed that isoniazid was capable of inhibiting LOX by competing for its obligatory cofactor, pyridoxal phosphate, and thus, regulated collagen deposition. Udupa (19) observed that isoniazid inhibits LOX activity and decreases tensile strength and collagen content. In addition, Udupa previously reported that isoniazid decreases the mechanical strength of collagen by inhibiting LOX and interfering with electrostatic interactions between collagen and the ground substance; which is important in wound healing and scar formation. Isoniazid has been shown to be a potent inhibitor of human fibroblasts at a concentration of 3 mg/kg and has been administered successfully in patients with lesions exhibiting a delayed healing process (20). Further studies are required to determine whether isoniazid suppresses granulation and scar tissue formation. This case study presents a novel potential application of isoniazid, however further investigation is required to demonstrate the importance of using isoniazid.

In conclusion, isoniazid reduces granulation tissue and prevents recurrent stenosis. However, further randomised control studies are required to investigate the possibility of interventional bronchology techniques combined with isoniazid treatment for the management of tracheobronchial obstructions. The administration of isoniazid may abate the requirement for repetitive bronchoscopies and invasive reconstructive surgery.
